# Lung Function Variation during the Estrus Cycle of Mares Affected by Severe Asthma

**DOI:** 10.3390/ani12040494

**Published:** 2022-02-17

**Authors:** Sophie Mainguy-Seers, Mouhamadou Diaw, Jean-Pierre Lavoie

**Affiliations:** Faculty of Veterinary Medicine, Department of Clinical Sciences, Université de Montréal, Saint-Hyacinthe, QC J2S 2M2, Canada; sophie.mainguy-seers@umontreal.ca (S.M.-S.); mouhamadou.diaw@umontreal.ca (M.D.)

**Keywords:** equine asthma, lung function, estrus cycle, progesterone, sex hormones

## Abstract

**Simple Summary:**

The estrus cycle and sex hormones influence asthma development and severity in humans, but whether the same is occurring in the asthma of horses is unknown. Severe equine asthma (SEA) is characterized by breathing difficulty, even at rest, and although it can be controlled by management and medication, it remains incurable. Stabling and hay feeding are the main contributors to disease exacerbation, but other factors could possibly alter the respiratory compromise. Therefore, the objective of this study was to evaluate the effects of the estrus cycle on airway dysfunction in five mares affected by SEA by assessing the lung function during the follicular and luteal phases of the reproductive cycle. The inspiratory obstruction improved during the luteal phase and the variation in progesterone and the dominant follicle size correlated with lung function parameters, suggesting a role for sex hormones in asthma pathophysiology. This first description of the estrus cycle’s modulation of airway obstruction in horses supports further studies to uncover the effects of sex hormones in asthma in horses and humans.

**Abstract:**

While the prevalence of asthma is higher in boys than in girls during childhood, this tendency reverses at puberty, suggesting an effect of sex hormones on the disease pathophysiology. Fluctuations of asthma severity concurring with the estrus cycle are reported in women, but this phenomenon has never been investigated in mares to date. The objective of this exploratory study was to determine whether the estrus cycle modulates airway obstruction in severe equine asthma (SEA). Five mares with SEA during exacerbation of the disease were studied. The whole breath, expiratory and inspiratory resistance, and reactance were compared during the follicular and luteal phases of the estrus cycle. The reproductive tract was evaluated by rectal palpation, ultrasound, and serum progesterone levels. The inspiratory resistance and reactance improved during the luteal phase of the estrus cycle, and variation in progesterone levels and the dominant follicle size correlated with several lung function parameters. The fluctuation of airway dysfunction during the estrus cycle is noteworthy as deterioration of the disease could perhaps be expected and prevented by horse owners and veterinarians. Further studies are required to determine if the equine species could be a suitable model to evaluate the effects of sex hormones on asthma.

## 1. Introduction

Studying the sex disparities in asthma pathophysiology is requisite to comprehending the influence of sex hormones to ultimately provide personalized care to asthmatic patients [1]. The higher prevalence of asthma in boys reverses at puberty, after which the disease becomes more prevalent in women [2]. Importantly, women are over-represented in some corticosteroid-resistant and severe asthma clusters [3,4], and the sex difference in hospitalization admissions are more pronounced during the reproductive years [5]. However, conflicting results obscure the precise influence of sex hormones in asthma. While some studies described a deterioration of respiratory symptoms or lung function during the perimenstrual phase or menses [6,7,8,9], when progesterone and estrogen levels are low, others have reported no variation during the estrus cycle [10]. Also, increased emergency visits for asthma have been described both in the pre-ovulatory [11,12] and perimenstrual periods [11].

Severe equine asthma (SEA) is a chronic respiratory disease affecting approximately 14% of adult horses living in a temperate climate [13], and it is mostly influenced by environmental conditions [14] and genetics [15]. A predisposition for females has been reported [16], but this is not a consistent finding [13], and whether the disease is altered by the estrus cycle is currently unknown. Therefore, the objective of this exploratory study was to determine if lung function differed during the luteal and follicular phases of the estrus cycle in mares affected by SEA. A significant improvement in the inspiratory obstruction was observed during the luteal phase of the estrus cycle in this study.

## 2. Materials and Methods

All experimental procedures were performed in accordance with the Canadian Council for Animal Care guidelines and were approved by the Animal Care Committee of the Faculty of Veterinary Medicine of the Université de Montréal on 27 May 2021 (Protocol # 21-Rech-2128). This manuscript follows the recommendations of the ARRIVE guidelines.

Six mares with SEA donated to a research herd were studied during the 2021 reproductive season in the northern hemisphere (between May and August). The mares were mixed breeds, aged 17.7 (16–21) years old and weighed 573 (509–664) kg. One mare had foaled in the past, 3 were never bred and the information was unknown for 2 mares. A priori power analysis was not performed as no data were available for the equine species for calculations. The diagnosis of SEA was based on a history of repeated periods of labored breathing at rest, abnormal lung function (transpulmonary pressure >15 cm H_2_O), and >25% neutrophils on bronchoalveolar lavage fluid (BALF) cytology when horses were stabled and fed hay, as previously recommended [17]. The mares had been part of the research herd for 2 to 6 years. At the beginning of the study, disease exacerbation was triggered by exposure to environmental antigens by stabling (wood shaving bedding) and dry hay feeding until respiratory efforts were visible at rest (median exposure of 36 days before the study). All mares had access to timed daily turnout on a dirt paddock, and the management remained the same for the duration of the experiment. The mares were conditioned to stand in a stock and to wear a mask. Endpoints included anorexia, decreased manure production, colic, hyperthermia, respiratory distress, or any other medical conditions that would have required treatment.

### 2.1. Pulmonary Function Tests

Lung function was evaluated with the Equine MasterScreen impulse oscillometry system (IOS; Jaeger GmbH, Würzburg, Germany) as previously described [18], in unsedated mares standing in stocks with the head in resting physiological position. Briefly, multi-frequency impulses produced by a loudspeaker were superimposed to the tidal breathing of the horse through an airtight mask. Simultaneously, pressure transducers connected to a pneumotachograph placed in front of the mask acquired the pressure-flow signal response of the respiratory system. The device was calibrated on each test day and accuracy was verified with a resistive test load before experimental measurements. Lung function data were acquired with LabManager (version 4.53, Jaeger, Würzburg, Germany) and analyzed with FAMOS (IMC, Meβsysteme, Berlin, Germany) using the fast Fourier transform method. Three 30 s recordings were averaged for analyses. Inspiratory (insp), expiratory (exp) and whole breath resistance (R), reactance (X), and coherence of the respiratory system from 2 to 7 Hertz (Hz) were analyzed. Briefly, the impulse of higher frequencies travels a shorter distance and thus represents changes occurring in central and upper airways, while lower frequency impulses travel deeper into the lungs where it detects peripheral airway dysfunction in diseases such as asthma [19].

### 2.2. Reproductive Tract Evaluation

After assessment of the lung function, the mares were sedated with xylazine (0.3 mg/kg IV) if needed, and the reproductive tract was examined by rectal palpation and ultrasound to determine whether the mares were in the follicular or the luteal phase of the estrus cycle by examining the cervix tonus, uterine edema, and the presence of relevant ovarian structures (major follicles and corpus luteum). The mares were scheduled to be evaluated every 7–10 days until an assessment of the lung function was obtained in the follicular and luteal phases, which was later confirmed by serum progesterone levels.

### 2.3. Serum Progesterone

Blood was collected in glass vacutainer tubes (BD Biosciences, Mississauga, Canada) before any other manipulations. After 20 min sedimentation, serum was obtained after centrifugation at 900× *g* for 10 min at room temperature, then stored at −80 °C until batch analysis. Serum progesterone levels were measured by chemiluminescence (IMMULITE, Siemens, Erlangen, Germany) by the Centre de diagnostic vétérinaire de l’Université de Montréal with a detection limit of 0.2 ng/mL. Results below the limit of detection were attributed the arbitrary value of 0.2 ng/mL for statistical comparisons. The values of ≤1 ng/mL and ≥5 ng/mL were considered representative of the follicular and luteal phases, respectively [20]. In instances where more than two serum progesterone levels were obtained in the same mare (when palpation and ultrasound gave ambiguous results and assessment was repeated at a later date within the same cycle), the lung function values at the time points with the lowest and highest progesterone levels were used for statistical analysis.

### 2.4. Statistical Analysis

Data were analyzed using GraphPad Prism version 8.4.3 for Windows (GraphPad Software, San Diego, CA, USA). Normality was assessed with Shapiro–Wilk tests. As lung function data were normally distributed, two-way ANOVA for repeated measures was used to evaluate the effects of the phase of the estrus cycle (follicular or luteal) and the IOS impulse frequency (from 2 to 7 Hz) on the lung function parameters. When a significant effect was observed with the ANOVA, Bonferroni multiple comparison tests were used to compare the data between the luteal and follicular phases at each frequency. Associations between the variation in lung function data, progesterone levels and the dominant follicle diameter were explored with Pearson or Spearman correlations as appropriate. Results are reported with mean, or with median if not normally distributed, and range for descriptive data and with 95% confidence interval (CI) for statistical results. Ambient conditions (temperature and humidity) were compared with paired t-tests, or Wilcoxon matched-paired signed rank tests, as appropriate.

## 3. Results

The temperature and humidity were not different between the evaluations in the luteal and follicular phases (respectively, a mean of 19.6 °C (16.4–22.9) and 19.4 °C (16.4–24.9), and a median of 45% (36–76) and 46% (36–84)).

### 3.1. Mares

Six mares were initially included in this study. In one mare, no reproductive tract was detected by rectal palpation or ultrasound on three occasions, a hymen persistence was present, and two serum progesterone levels taken at a 12-day interval were under the limit of detection. Combined, these findings suggested a congenital developmental anomaly, and data from that mare were excluded. The other five mares had normal reproductive tracts, based on clinical and ultrasonographic examinations.

The mean progesterone level was 9.4 (5.0–13.2) and 0.5 (<0.2–1.4) ng/mL in the luteal and follicular phases, respectively. The mare with the nadir of progesterone of 1.4 ng/mL was still included based on an eight-fold difference with the progesterone value during the luteal phase [21].

### 3.2. Lung Function Variation

The exacerbation of the disease was confirmed at the first lung function assessment (ratio of the resistance at 3 and 7 Hz (R3/R7) ≥1 and negative reactance from 2–7 Hz). Three mares had their first evaluation during the follicular phase, and two during the luteal phase.

#### 3.2.1. Whole-Breath Analysis

**Figure 1:**
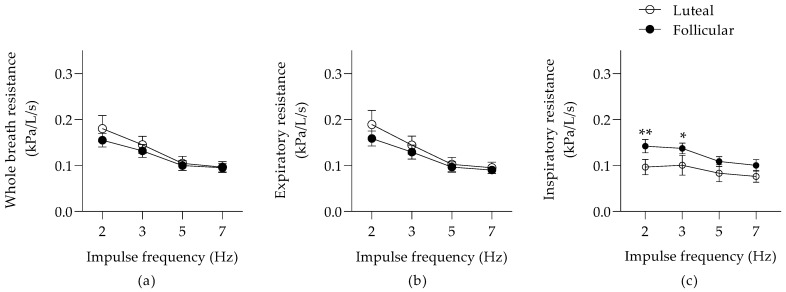
Pulmonary resistance (means ± SEM) during whole-breath (**a**), expiration (**b**), and inspiration (**c**). * *p* < 0.05 and ** *p* < 0.01 between the estrus phases with Bonferroni’s multiple comparison tests.

**Figure 2:**
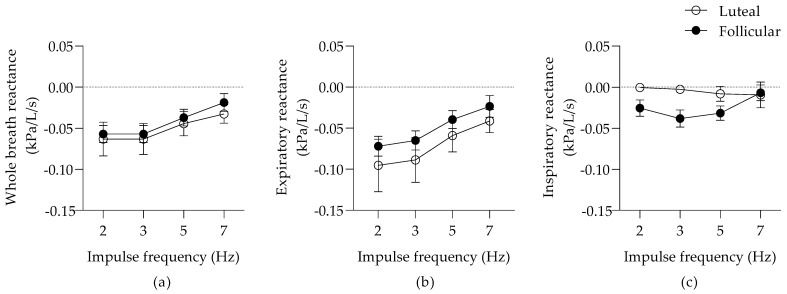
Pulmonary reactance (means ± SEM) during whole-breath (**a**), expiration (**b**), and inspiration (**c**).

There was no significant effect of the phases of the estrus cycle on the whole-breath R and X (Figure 1a and Figure 2a). The whole-breath R was frequency-dependent (*p* = 0.009), and a similar trend was present for the whole-breath X (*p* = 0.06).

#### 3.2.2. Within-Breath Analysis

There was a significant frequency dependence of Rexp (*p* = 0.005) and Xexp (*p* = 0.047), but no effect of the estrus cycle during expiration (Figure 1b and Figure 2b). At inspiration, there was a significant effect of the estrus cycle on the pulmonary Rinsp (*p* < 0.0001; Figure 1c) and Xinsp (*p* = 0.016; Figure 2c). The Rinsp at 2 and 3 Hz were improved during the luteal phase (a mean decrease of 0.046 (95% CI; 0.012–0.079) kPa/L/s and 0.037 (95% CI; 0.004–0.07) kPa/L/s, respectively).

#### 3.2.3. Coherence

**Figure 3:**
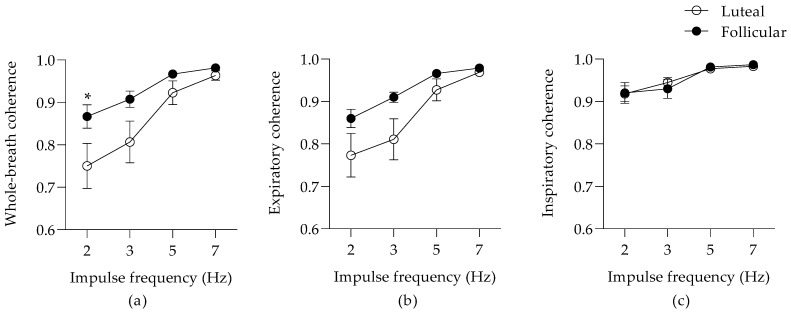
Coherence (means ± SEM) during whole-breath (**a**), expiration (**b**), and inspiration (**c**). * *p* < 0.05 between the estrus phases with Bonferroni’s multiple comparison tests.

The coherence represents the causality between the flow input and the resultant pressure signals of the respiratory system and is an indicator of the accuracy of the mathematical model to predict the impedance data. Although it may be used as a quality control in oscillometry, coherence is also influenced by the severity of the disease itself [22]. The whole-breath (*p* = 0.0006; Figure 3a) and expiratory (*p* = 0.0002; Figure 3b) coherence were frequency-dependent and were both influenced by the estrus cycle (respectively, *p* = 0.002 and *p* = 0.008). There was a significant decrease of the whole-breath coherence at 2 Hz during the luteal phase (a mean reduction of 0.117 (95% CI; 0.014–0.219)). The inspiratory coherence was influenced by the impulse frequency (*p* = 0.0003; Figure 3c), but not by the estrus cycle.

### 3.3. Correlations

**Figure 4:**
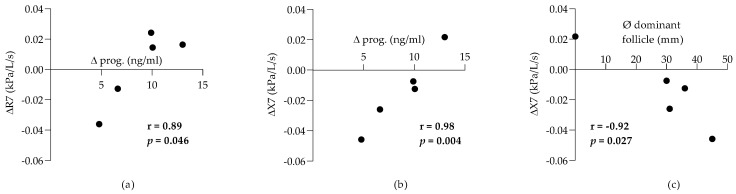
Correlations between the variation in progesterone (prog.) and the variation in pulmonary resistance (R) at 7 Hz (**a**), the variation of pulmonary reactance (X) at 7 Hz (**b**). Correlation between the diameter (Ø) of the dominant follicle in the follicular phase and variation in pulmonary X at 7 Hz (**c**).

The variation (difference between the luteal and follicular values) of serum progesterone was positively correlated with the variation of whole-breath R (r = 0.89, *p* = 0.046; Figure 4a), expiratory R (r = 0.94, *p* = 0.017), and X (r = 0.98, *p* = 0.004; Figure 4b) at 7 Hz. These results indicate that increasing progesterone was associated with a worsening of the resistance but an improving reactance. Correlations between the variation in lung function (the difference between the luteal and follicular values) and the diameter of the dominant follicle during the follicular phase were also assessed, as systemic estrogen has been shown to strongly correlate with the size of the dominant follicle in mares [23]. The diameter of the dominant follicle was negatively correlated with the variation of the whole-breath X at 3 Hz (r = −0.90, *p* = 0.036), 5 Hz (r = −0.97, *p* = 0.006), and 7 Hz (r = −0.92, *p* = 0.027; Figure 4c), and with the variation of Xexp at 3 Hz (r = −0.90, *p* = 0.04) and 5 Hz (r = −0.95, *p* = 0.012). It was correlated positively with the Rexp at 2 Hz (r = 0.89, *p* = 0.044). These results show that the size of the dominant follicle was associated with an improvement in the reactance and in the small airway’s resistance during the follicular phase.

## 4. Discussion

This study shows that the estrus cycle alters the lung function of mares with SEA, with an improvement in the inspiratory resistance and reactance during the luteal phase. These findings, combined with the correlations between variation in progesterone levels and the dominant follicle size with lung function parameters, suggest an influence of sex hormones on asthma pathophysiology in horses.

### 4.1. Estrus Cycle, Sex Hormones, and Lung Function

Asthma in humans and horses is characterized by expiratory flow limitation [24]. Expiratory parameters are indicative of intra-thoracic, thus smaller airway function, while inspiratory data represents predominantly extra-thoracic airways. Therefore, the improvement of inspiratory resistance during the luteal phase observed in the current study suggests a change of airway caliber occurring in the upper or most central airways [25]. Interestingly, the genioglossus muscle, an important upper airway dilator, has increased activity in women during the luteal phase [21], and synthetic progestins can improve inspiratory dysfunction during sleep apnea in humans, a disorder characterized by upper airway obstruction [26]. As the genioglossus muscle is also deemed important for stabilization of the equine pharynx [27], it is possible that modulation of its activity during the estrus cycle contributed to the inspiratory modifications observed in the current study. Alternatively, variations in lung function might be more easily detected during inspiration as the expiratory phase is much more compromised in asthma. As such, selected inspiratory parameters are better indicators of day-to-day variation in lung function in asthmatic adolescents [28].

The variation in serum progesterone levels between the luteal and follicular phases was strongly positively correlated with the difference in whole-breath and expiratory R and X at 7 Hz, indicating an association between progesterone and worsening resistance but improving reactance. These results could be caused by opposing actions of this hormone on separate airway compartments. Progesterone induces relaxation of the urogenital tract smooth muscle, and a similar effect is expected for airway smooth muscle (ASM) [29], which could explain the improved reactance. However, ASM relaxation would normally also improve pulmonary resistance, at least if it was occurring in the central airways. The tidal volume is higher during the luteal phase in healthy women when progesterone levels are high, compared to the follicular phase [30]. If a similar modification of tidal volume was present in mares, it could contribute to the association between improving reactance and progesterone. A decrease in peribronchial collagen deposition, reported after progesterone administration in mice, could also improve reactance [31]. These beneficial effects of progesterone could be relevant clinically, as suggested by the stabilization of peak flow rate after its administration in women with life-threatening exacerbation of asthma during the premenstrual phase [32]. Other properties of progesterone could explain its association with increasing airflow resistance, such as a decrease in epithelial ciliary beat frequency [33] and central hyperventilation [34]. Indeed, hyperventilation has been shown to increase pulmonary resistance as assessed by oscillometry in healthy horses [35]. Of note, progesterone receptors exist in two isoforms (A and B) and each mediates different biological activities [36]. Determining the proportion and the localization of these receptors in the equine respiratory tract is necessary to uncover the distinctive effects of progesterone on lung function observed in this study.

The size of the dominant follicle was positively correlated with the variation in whole-breath reactance at 5 Hz and the expiratory reactance at 5 and 7 Hz, suggesting positive effects of estrogen during the follicular phase [23]. Estrogen relaxes the ASM [37,38], decreases the proliferation of lung myofibroblasts [39], and enhances the effects of β2-adrenergic agonists in vitro [29]. In rodent experimental models, estrogen reduces airway hyperresponsiveness [40,41] and lung inflammation [41] in ovariectomized animals, and the absence of the estrogen receptor-α (knockout mice) is associated with lung dysfunction [42]. While estrogen has been shown to increase mucus production in vitro [43], the contrary was observed in a mouse model of allergic asthma [41]. Despite these interesting properties in experimental conditions, the clinical effects of estrogen in women are difficult to delineate and often contradictory. Its administration improves asthma symptoms in women in some studies [9], but not in all [44]. Its levels are negatively associated with lung function in adolescents [45], and the use of hormonal replacement therapy is related to increased odds of new-onset asthma after menopause [46]. Furthermore, single nucleotide polymorphisms of the estrogen receptor α are associated with a decline in lung function in asthmatics, particularly in women [47]. Taken together, these results suggest both beneficial and deleterious impacts of estrogen, and its actions might vary depending on the physiological status of the patients. In mares, the biological effects of estrogen on airway cells, the distribution and proportion of each receptor subtypes (α and β) along the respiratory tract, and the influence of endogenous or exogenous estrogen in SEA have not been studied to date. However, tamoxifen, a synthetic selective estrogen receptor modulator, induces a mild reduction of airway resistance in horses with SEA, suggesting a possible effect of this sex hormone [48].

Estrogen and progesterone could act synergistically to modulate lung function. The isometric contraction of mouse tracheal rings is more strongly attenuated by a combination of estrogen and progesterone, in concentrations representative of levels observed during human pregnancy, compared to the sole effect of each hormone [38]. Furthermore, both estrogen and progesterone levels correlated positively with the peak expiratory flow in a woman with perimenstrual asthma [49]. These findings would fit well with the data from the current study, which suggests that both hormones are associated with an improvement in reactance. This would also be consistent with anecdotal reports of improvement of respiratory signs during the gestation of mares with SEA (personal communication) and during pregnancy in some women [50].

### 4.2. Limitations and Areas of Future Research

The main limitation of this study was the assessment of a low number of mares during only one estrus cycle. Ideally, future investigations should follow larger cohorts during multiple cycles to obtain a more precise understanding of the phenomenon. The mares included in this study were aged from 16 to 21 years old, which is expected as aging is associated with an increased risk of SEA [13,16]. However, the estrus cycle is also influenced by aging in mares [51,52], with hormonal modifications and subfertility starting in the teens and culminating in the cessation of ovarian activity around 25 years old [52]. Therefore, future studies should ideally include mares within a wider age range. Additionally, as the effects of the estrus cycle on the lung function in healthy mares are unknown, a control group of healthy mares and of males with SEA should be included in future studies.

The effects of other hormones that could influence asthma physiology, such as testosterone [53,54] and sex hormone-binding globulin [55], were not investigated in this study, and estrogen levels were not directly measured. Interestingly, endogenous cortisol was shown to vary during the estrus cycle, with a higher value during the mid-late luteal phase in pony mares [56]; however, this was not observed by others [57]. Perhaps a variation of endogenous glucocorticoids could have contributed to the modulation of lung function in the current study, and this should be explored in future experiments. Furthermore, other factors that mediate sex differences in asthma, such as the smaller airway caliber in women [2], have never been investigated in horses to our knowledge.

Finally, the data from 2–7 Hz were reported in this study because the variation between recordings was low at these frequencies (mostly <15% [22]), lower frequencies better described the dysfunction in SEA [58], and coherence values were acceptable. The frequency dependence of the expiratory resistance and reactance is typical of small airway disease and has been previously reported by a forced oscillometry technique in SEA [58]. Additionally, the frequency-dependence of the coherence was not surprising as more severe lung dysfunction in smaller airways is expected during exacerbation. However, the cause of the poorer coherence at lower frequencies during the luteal phase for whole-breath and expiratory parameters in the current study is unknown. It might be related to increased heterogeneity of the respiratory system, perhaps suggesting a worsening of expiratory lung dysfunction during the luteal phase. Artifacts during measurements, such as leaks, swallowing, and coughing, could also result in lower coherence, but care was taken to repeat recording when such an event occurred. As measurements were performed concurrently for luteal and follicular assessment in different mares, the difference in coherence related to the estrus cycle is unlikely to be caused by a technical variation. Importantly, the differences in R and X during the estrus cycle occurred during the inspiratory phase, which had high coherence values that were not influenced by the phase of the estrus cycle and removing the few data with low coherence values (0.6–0.7) did not modify the results. Confirming the variation of lung function through the estrus cycle with standard lung function would be relevant, but given the current results showing differences only with within-breath analysis, it is unlikely that pleural pressure measurements would have the sensitivity required to detect these changes.

## 5. Conclusions

This study describes for the first time the influence of the estrus cycle on the natural course of SEA and supports further investigations to determine if horses could be a relevant model to explore the roles of sex hormones in asthma. Indeed, the reproductive physiology of mares and women is similar in many aspects, including the prolonged follicular phase and the monovolution [52]. The ease of reproductive tract evaluation and the long life span of horses, compared to rodent models, are additional valuable features [52]. Even the seasonality of reproduction in mares represents an opportunity to assess the effects of exogenous sex hormones when estrus activity is null. To further delineate the significance of the current results, future investigations should examine the localization and proportion of progesterone and estrogen receptors in the equine respiratory tract and the effects of these sex hormones on the biology of airway cells.

## Data Availability

The datasets generated in the current study are available in the Dataverse UdeM repository (https://doi.org/10.5683/SP3/XGYUKU).
